# Pharmacological Blockade of Adenosine A_2A_ but Not A_1_ Receptors Enhances Goal-Directed Valuation in Satiety-Based Instrumental Behavior

**DOI:** 10.3389/fphar.2018.00393

**Published:** 2018-04-24

**Authors:** Yan Li, Xinran Pan, Yan He, Yang Ruan, Linshan Huang, Yuling Zhou, Zhidong Hou, Chaoxiang He, Zhe Wang, Xiong Zhang, Jiang-Fan Chen

**Affiliations:** ^1^Department of Neurology, The Second Affiliated Hospital and Yuying Children’s Hospital of Wenzhou Medical University, Wenzhou, China; ^2^School of Optometry and Ophthalmology and Eye Hospital, The Institute of Molecular Medicine, Wenzhou Medical University, Wenzhou, China; ^3^Department of Neurology, School of Medicine, Boston University, Boston, MA, United States

**Keywords:** adenosine A_2A_ receptor, adenosine A_1_ receptor, goal-directed behavior, habit, instrumental behavior

## Abstract

The balance and smooth shift between flexible, goal-directed behaviors and repetitive, habitual actions are critical to optimal performance of behavioral tasks. The striatum plays an essential role in control of goal-directed versus habitual behaviors through a rich interplay of the numerous neurotransmitters and neuromodulators to modify the input, processing and output functions of the striatum. The adenosine receptors (namely A_2A_R and A_1_R), with their high expression pattern in the striatum and abilities to interact and integrate dopamine, glutamate and cannabinoid signals in the striatum, may represent novel therapeutic targets for modulating instrumental behavior. In this study, we examined the effects of pharmacological blockade of the A_2A_Rs and A_1_Rs on goal-directed versus habitual behaviors in different information processing phases of instrumental learning using a satiety-based instrumental behavior procedure. We found that A_2A_R antagonist acts at the coding, consolidation and expression phases of instrumental learning to modulate animals’ sensitivity to goal-directed valuation without modifying action-outcome contingency. However, pharmacological blockade and genetic knockout of A_1_Rs did not affect acquisition or sensitivity to goal-valuation of instrumental behavior. These findings provide pharmacological evidence for a potential therapeutic strategy to control abnormal instrumental behaviors associated with drug addiction and obsessive-compulsive disorder by targeting the A_2A_R.

## Introduction

Goal-directed and habitual behaviors are crucial adaptive behaviors for our daily life. Goal-directed behavior evaluates actions prospectively and can flexibly adjust action depending on environmental changes, but this comes at the cost of more cognitive resource. By contrast, habitual behavior is usually developed after repeated overtraining for days and represents automatic responses elicited by external or internal triggers during the performance of routine procedures with less cognitive loads (Dolan and Dayan, 2013). These two behavioral processes can develop in parallel or sequentially and can also reciprocally compete with each other for behavioral control (Yin and Knowlton, 2006; Balleine and O’Doherty, 2010; Kim and Hikosaka, 2015). The balance between flexible goal-directed actions and repetitive habitual behaviors has an essential role in achieving optimal performance of behavioral task. Dysregulation of goal-directed versus habitual behaviors is considered to be a potential mechanism underlying the relapse of drug addiction (Ostlund and Balleine, 2008), obsessive compulsive disorder (Gillan et al., 2011; Robbins et al., 2012; Burguiere et al., 2015), and may contribute to the executive dysfunction in Parkinson’s (Redgrave et al., 2010; de Wit et al., 2011) and Huntington’s disease patients (Lawrence et al., 1998).

The striatum plays an essential role in control of goal-directed versus habitual behaviors (Yin and Knowlton, 2006; Graybiel and Grafton, 2015; Kim and Hikosaka, 2015). The dorsal medial striatum (DMS)-connecting orbitofrontal cortex (OFC) is critical for goal-directed valuation (Gremel and Costa, 2013), while the dorsal lateral striatum (DLS) and its connecting infralimbic cortex act as dual operators for habitual behavioral control (Smith and Graybiel, 2013a,b). Additionally, the accumbens nucleus (NAc)-ventral Pallidum (VP) pathway is necessary for goal-directed valuation as inactivation of NAc-VP pathway impairs the predictive learning (Leung and Balleine, 2013). Furthermore, the nigro-striatal dopamine signaling acts as a prediction error and motivational signal to drive instrumental learning (Glimcher, 2011; Rossi et al., 2013; Steinberg et al., 2013). Thus, the striatum acts as a key locus in integrating the cortico-striatal glutamate and the substantia nigra-striatal dopamine signals to control goal-directed and habitual behaviors.

The striatal control of instrumental behaviors is accomplished through a rich interplay of the numerous neurotransmitters and neuromodulators to modify the input, processing and output functions of the striatum (Lovinger, 2010). Several studies have documented the involvement of the D_2_ receptor (Kwak et al., 2014), cannabinoid receptor type 1 (CB_1_R) (Hilario et al., 2007) and 5-hydroxytryptamine 6 (5-HT_6_) receptor (Eskenazi et al., 2015) in control of instrumental behavior. However, pharmacological control of instrumental behaviors is under-explored and the effective pharmacological strategies for the control of goal-directed versus habitual behaviors are lacking. Adenosine A_1_ and A_2A_ receptors are highly expressed in the striatum and are increasingly recognized as important pharmacological targets for controlling cognition under normal and disease conditions (Chen et al., 2013; Chen, 2014). The Gs-coupled facilitating A_2A_ receptor (A_2A_R) and Gi-coupled inhibitory A_1_ receptor (A_1_R) both integrate dopamine (Shen W. et al., 2008), glutamate (Kreitzer and Malenka, 2007), and BNDF (Tebano et al., 2008; Wei et al., 2014) signaling to modulate synaptic plasticity and control cognition. For example, using our newly developed chimeric rhodopsin-A_2A_R proteins (optoA_2A_R), we recently demonstrated that transient activation of A_2A_R by light in a time-locked manner with reward delivery is sufficient to impair goal-directed behavior whereas focal knockdown of A_2A_R in the striatum enhances goal-directed behaviors (Yu et al., 2009; Li et al., 2016). Similarly, pharmacological blockade of A_2A_R promoted goal-directed seeking for ethanol in ENT1 knockout mice (Nam et al., 2013b) and restored goal-directed sensitivity to negative feedback in the methamphetamine (METH)-paired context (Furlong et al., 2017). These pharmacological, genetic, and optogenetic demonstrations of the cognitive “brake” mechanism of A_2A_R activation led us to propose that pharmacological blockade of the A_2A_R represents a promising therapeutic target for controlling goal-directed behaviors.

As the first step in developing an adenosine receptor-based pharmacological approach to control the goal-directed versus habitual behaviors, we coupled the A_2A_R antagonist (KW6002) and A_1_R antagonist (DPCPX) with the satiety-based instrumental learning paradigm to address the effect of pharmacological blockade of the A_2A_R and A_1_R on three aspects of instrumental learning processes: (i) behavioral elements of instrumental behaviors (i.e., acquisition of action-outcome contingency versus goal-evaluation) by acquisition of instrumental behavior, the devaluation test and the omission test; (ii) the instrumental learning processes by administering the A_2A_R antagonist either prior to the training (learning/encoding) or post-training (consolidation) during the random interval (RI) schedule, or immediately before the devaluation and omission tests (expression/retrieval of instrumental behaviors); (iii) the potential role of the A_1_ receptor in control of instrumental learning.

## Materials and Methods

### Animals

Animals were handled in accordance with the protocols approved by the Institutional Ethics Committee for Animal Use in Research and Education at Wenzhou Medical University, China. C57BL/6 male mice at least 8 weeks old (23–27 g each) were used in the experiments. The A_1_R knockout mice (A_1_R^-/-^=^+/+^) and wild-type littermate controls (A_1_RC=C) have been well characterized previously (Johansson et al., 2001) and confirmed by PCR analysis of gene identification before the experiment. Mice were housed in an ambient temperature of 22 ± 0.5°C and a relative humidity of 60 ± 2% with a 12 h light/dark cycle. Mice were single-housed and underwent experiments in the light cycle.

### Satiety-Based Instrumental Training and Testing

**FIGURE 1:**
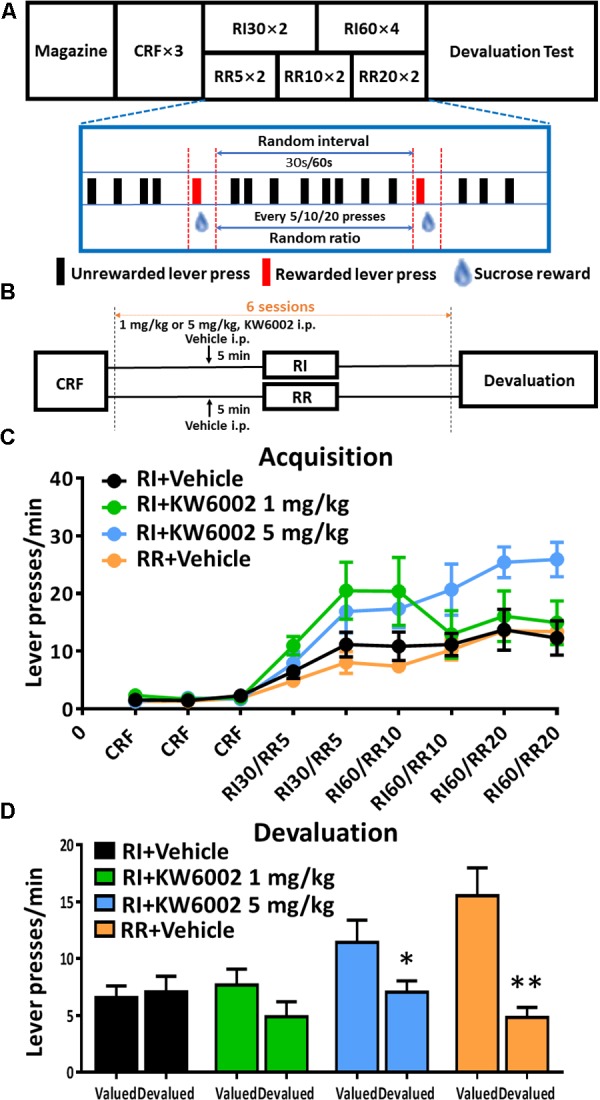
Pharmacological blockade of A_2A_Rs promoted goal-directed valuation. **(A)** Satiety-based instrumental behavior design schematic. Mice underwent Magazine-CRF-RI/RR-Devaluation procedure sequentially. CRF, continuous reinforcement; RI, random interval; RR, random ratio. **(B)** KW6002 and vehicle were injected intraperitoneally 5 min before daily RI training session at different doses (1 and 5 mg/kg), meanwhile vehicle was administrated 5 min before daily RR training session as another control group to form goal-directed behavior **(C)**. All mice gradually increased their lever presses in the RI/RR training sessions (training main effect: *p* < 0.001). There was the interaction effect of training sessions X drug administration groups (*p* = 0.006) and between subject effect of different drug administration groups (*p* = 0.022). The statistical significance was only observed between RI+KW6002 5 mg/kg and RR + Vehicle groups (*post hoc* by Bonferroni test, *p* = 0.035). **(D)** In the devaluation test, mice trained with RI and RR procedures performed habitual (*p* = 0.755) and goal-directed (*p* = 0.002, ^∗∗^*p* < 0.01) behaviors, respectively, as designed. Mice received 1 mg/kg KW6002 tended to decrease their lever presses in the devalued condition but with no statistical significance (*p* = 0.141), while mice of 5 mg/kg group displayed markedly goal-directed performance in the devaluation test (*p* = 0.030, ^∗^*p* < 0.05). All data was analyzed by two-way ANOVA for repeated measurement, followed by *post hoc* comparison with Bonferroni test [RI group, *n* = 8; RI+KW6002 (1 mg/kg) group, *n* = 7; RI+KW6002 (5 mg/kg) group, *n* = 8; RR group, *n* = 9].

All instrumental learning experiments were performed in standard operant chambers (Med Associates). Each chamber was equipped with a retractable lever on either side of a pump with a syringe that delivered liquid reward (20% sucrose solution, 20 μl/reinforce which can be suspended from the syringe) and a house light (3 W, 24 V) mounted on the opposite side of the chamber. Training and testing procedures were performed following Rossi et al (Rossi and Yin, 2012) and illustrated in **Figure 1A**. In brief, mice were first given one 30-min magazine training session during which the sucrose solution was delivered on a random time 60 s schedule with the lever removed. Three days of continuous reinforcement (CRF) training sessions were followed to sufficiently establish the initial association between lever press and reward. At the start of the session, the house light was illuminated, and one lever was inserted into the chamber. The house light remained illuminated and the lever remained inserted and active during the entire session. During CRF session, each lever press resulted in the delivery of one drop of 20 μl 20% sucrose solution. Sessions ended after 60 min or when 50 rewards had been earned, whichever came first. After CRF, mice underwent RI schedule which was critical for habitual learning. They were trained 2 days on RI 30 s, with a 0.1 probability of reward availability every 3 s contingent upon lever pressing, followed by 4 days on the 60 s interval schedules (0.1 probability of reward availability every 6 s contingent upon lever pressing). Just as CRF training, RI sessions ended after 60 min or when 50 rewards had been earned, whichever came first. To further confirm goal-directed behavioral pattern, we also employed random ratio (RR) training paradigm as control which contributed to goal-directed behavior. Progressively leaner schedules of reinforcement were used: CRF for 3 days, then RR 5 for 2 days (RR5; each response was rewarded at a probability of 0.2 on average), RR10 for 2 days and finally RR20 for 2 days. In the training sessions, home chows were given 1.5–2g daily to maintain 80–85% of their free-feeding weight.

Following the RI/RR training sessions, a 2-day devaluation test was conducted. A specific satiety procedure was applied to alter the current value of a specific reward. On each day the mice were allowed to have free access to home chows, which were used for maintaining their weights in the training sessions or sucrose solution which was earned by their lever pressing for at least an hour to achieve sensory-specific satiety. Immediately after the unlimited pre-feeding session, mice were given a 5-min extinction test during which the lever was inserted and pressing times were recorded without reward delivery. The order of the valued and devalued condition tests (day 1 or day 2) was counterbalanced across animals. Mice sensitive to manipulation of outcome value would significantly reduce their lever presses on the devalued condition compared with the valued condition. Then after two supplementary RI60 training sessions, mice were further evaluated by a 30-min omission test in which action-outcome contingency was altered. In the omission test, mice had to control their lever-press impulsion formed by previous training sessions for 20 s to obtain the reward. Any lever press would reset the time counter and mice would hold another 20 s not to press the lever for reward delivery.

### Drug Administration

**FIGURE 2:**
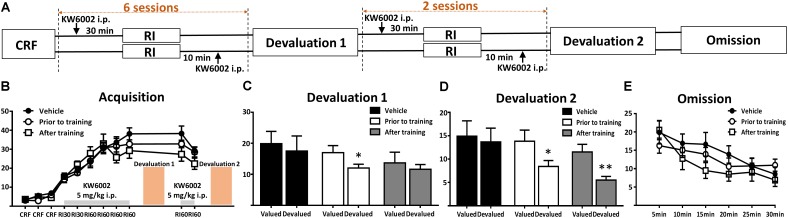
Pharmacological blockade of A_2A_Rs prior to and post daily training session promoted goal-directed seeking but not acquisition of instrumental conditioning. **(A)** Experimental design schematic with KW6002 injected intraperitoneally prior to and post-training. **(B)** There was no significant difference in acquisition of instrumental learning among these groups for lack of between groups effect (*p* = 0.593) and training X drug administration groups interaction effect (*p* = 0.108). **(C)** In the first devaluation test, mice with KW6002 injected prior to training showed sensitive to outcome devaluation (*p* = 0.021, ^∗^*p* < 0.05), compared to vehicle (*p* = 0.223) and that with KW6002 treated post-training (*p* = 0.539). **(D)** Then after two additional days of RI60 training, whatever KW6002 administered prior to (*p* = 0.034, ^∗^*p* < 0.05) or post (*p* = 0.008, ^∗∗^*p* < 0.01) training, mice displayed sensitive to outcome devaluation in the second devaluation test compared to the vehicle group (*p* = 0.482). **(E)** All mice decreased their lever presses indistinctively in the omission test in which the action-outcome contingency was reversed, showing neither testing time X drug administration groups interaction effect (*p* = 0.359) nor between-subject effect of drug administered groups (*p* = 0.836). All data was analyzed by two-way ANOVA for repeated measurement, followed by *post hoc* comparison with Bonferroni test (*n* = 8/group).

**FIGURE 3:**
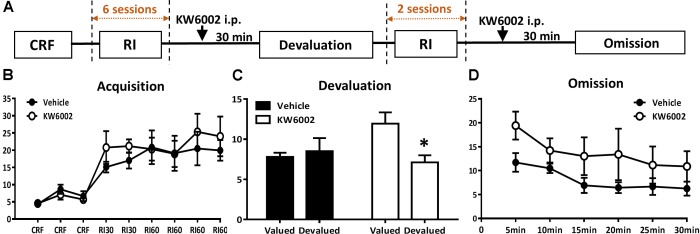
Pharmacological blockade of A_2A_Rs specifically in the expression phase of instrumental conditioning selectively promote goal-directed valuation but not action-outcome contingency. **(A)** Experimental design schematic with KW6002 injected intraperitoneally in the expression phase (i.e., devaluation and omission test) of instrumental behavior but not available in the training sessions. **(B)** Mice established instrumental conditioning indistinctively in the acquisition phase without between pre-manipulation groups effect (*p* = 0.541) and interaction effect of training sessions X pre-manipulation groups (*p* = 0.608). **(C)** KW6002 5 mg/kg or vehicle was administered 30 min before reward/home chow condition (i.e., devalued/valued condition). After 1-h exposure to devalued/valued condition at liberty, the devaluation test was proceeded in which reward delivery was absent and lever presses was recorded. Mice with KW6002 injected performed more goal-directed (*p* = 0.017, ^∗^*p* < 0.05), compared to that injected with vehicle (*p* = 0.710). **(D)** After 2-day extended RI60 training sessions, KW6002 5 mg/kg or vehicle was injected 30 min before omission test. Mice of both groups significant decreased their lever presses (time main effect, *p* = 0.020). But there was neither between-subject effect of drug treatments (*p* = 0.089) nor drug treatments X testing time interaction effect (*p* = 0.728). All data was analyzed by two-way ANOVA for repeated measurement, followed by *post hoc* comparison with Bonferroni test (vehicle group, *n* = 8; KW6002 group, *n* = 7).

The following drugs were used in the present study: KW-6002 ((E)-1,3-diethyl-8-(3,4-dimethoxystyryl)-7-methyl-3,7-dihydro-1H-purine-2,6-dione, a selective adenosine A_2A_R antagonist) and DPCPX (8-cyclopentyl-1,3-dipropylxanthine, a selective adenosine A_1_R antagonist). KW-6002 (1 mg/kg, 5 mg/kg, Sundia, United States) was suspended in dimethyl sulfoxide (DMSO, sigma), ethoxylated castor oil (Sigma) and water with a proportion of 15%:15%:70%. DPCPX (6 mg/kg, Abcam) was dissolved in 0.9% NaCl with 5% DMSO. The control mice were treated with corresponding vehicles. All the solutions were prepared immediately before administration. The administered doses of KW-6002 and DPCPX referred to previous researches (Chen et al., 2001; Prediger et al., 2004; Nguyen et al., 2014). Drugs were injected intraperitoneally (i.p.) routinely in a volume of 0.1 ml/10 g of body weight. The specific drug administration time course depended on experimental designs: prior to (30 min before) and post (10 min after) everyday RI training for learning and consolidation periods of instrumental learning, respectively (**Figure 2A**), while treated 30 min before devaluation test/omission test, but not available in the RI training sessions for expression of instrumental behavior (**Figure 3A**).

### DPCPX Concentration Detection

Considering the critical role of the striatum in control of instrumental behavior, we measured the concentration of DPCPX in the striatum of mice after intraperitoneal injection to verify the effective concentration of DPCPX. 30 min after DPCPX (6 mg/kg, i.p.) administration, the striata of mice were collected and homogenized. 0.1 ml of collected homogenate was added to a 1.5 ml centrifuge tube and followed by the addition of 0.01 ml methanol and 0.3ml of acetonitrile. The tubes were vortex mixed for 0.5 min. After centrifugation at 13,000 rpm for 10 min, 100 μl of supernatant was transferred to an auto-sampler vial. Next, 2 μl of the mixture was injected into the LC-MS/MS system for analysis. DCPCX concentrations were determined by ultrahigh performance liquid chromatography with mass spectrometry method (UHPLC-MS/MS). UHPLC-MS/MS analyses were performed by an Agilent UHPLC unit (Agilent Corporation, MA, United States) with a ZORBAX Eclipse Plus C18 column (1.8 μm, 2.1 × 50 mm, I.D. Agilent Corporation, MA, United States) thermostated at 25°C. The mobile phase was composed of 0.1% formic acid (A) and acetonitrile (B) with gradient as follows: 0.0 min at 50% B, 0.0–2.0 min linear increase to 98% B, and 2.0–3.5 min at 50% B and the flow rate was 0.4 ml/min. The total run time was 3.5 min. The electrospray interface was maintained at 500°C. Nitrogen nebulization was performed with a nitrogen flow of 800 l/h. Argon was used as the collision gas. DPCPX was detected in multiple reaction monitoring (MRM) scan mode with positive ion detection. The precursor-product ion pairs used for the MRM detection were m/z 305.4 → 178.1 for DCPCX.

### Quantitative PCR of A_1_R mRNA

Striatal tissues from A_1_R KO mice and their WT littermates were analyzed by the quantitative real-time polymerase chain reaction (qPCR) procedure as we have described previously (Zhang et al., 2015) using the following forward and reverse primers for A_1_R mRNA: primers: forward, 5′-CATCCTGGCTC TGCTTGCTATT-3′; reverse and 5′-TTGGCTATCCAGGCTTGTTCC-3′.

### Statistical Analysis

All data presented as mean ± SEM and were processed with SPSS 17.0. Two-way ANOVA for repeated measurements was used with training/testing sessions as within-subject effect and different drug administrations/genotypes as between-subject effect, followed by *post hoc* comparison by Bonferroni test, and with *p* < 0.05 as statistical significance.

## Results

### Pharmacological Blockade of A_2A_Rs Promoted Goal-Directed Valuation

To perform flexible, goal-directed actions, animals must acquire the ability to encode both the *contingency* between a specific action and its outcome, and the current *value* of the outcome during instrumental conditioning (Balleine and Dickinson, 1998). We administered KW6002 (i.p. at 1 mg/kg or 5 mg/kg or vehicle) 5 min prior to everyday RI training session which was critical for establishment of habitual action (**Figure 1B**) to investigate the modulatory effect of A_2A_R blockade on the acquisition of instrumental behaviors. To better identify goal-directed behavioral pattern, we have also included another group of mice that were trained in parallel with RR paradigm which led to goal-directed behavior as control (**Figure 1B**). All mice gradually increased their lever presses and reached a platform eventually, indicating the successful training paradigm (**Figure 1C**). Mice treated with KW6002 at 5 mg/kg significantly elevated lever presses rate (interaction effect of training sessions X drug administration groups: *F*_5,140_ = 2.659, *p* = 0.006; between-subject effect of drug administration groups: *F*_3,28_ = 3.740, *p* = 0.022): the statistical significance was observed between the RI + KW6002 5 mg/kg and the RR + Vehicle groups (Bonferroni *post hoc* test, *p* = 0.035) but absent in any other comparison pairs including RI+KW6002 5 mg/kg versus RI + Vehicle groups (*post hoc* by Bonferroni test, *p* = 0.116).

The outcome devaluation procedure was used to demonstrate the importance of the evaluative components of goal-directed actions by A_2A_R blockade. In the devaluation test, lever presses rates between the valued and devalued conditions were compared (**Figure 1D**). Mice in the RI + Vehicle training group did not decrease lever presses in the devalued condition, showing no devaluation effect and indicating a habitual behavior (*F*_1,7_ = 0.105, *p* = 0.755), while the RR + Vehicle training group significantly decreased their lever presses (*F*_1,8_ = 20.865, *p* = 0.002), demonstrating goal-directed behavior. Notably, KW6002 at 1 mg/kg tended to decrease lever pressing rate in devalued condition compared to valued condition (*F*_1,6_ = 2.867, *p* = 0.141), whereas KW6002 at 5 mg/kg group showed markedly sensitive to outcome devaluation with decreased level pressing rate (*F*_1,7_ = 7.418, *p* = 0.030). Thus, pharmacological blockade of A_2A_R promoted goal-directed valuation. Whether the A_2A_R antagonist influence the acquisition of the instrumental learning need further clarification since the increased lever presses rate by KW6002 in the acquisition phase might be attributed to the improvement in instrumental learning or enhanced general motor activity effect of the A_2A_R antagonist given the drug administration immediately (∼5 min) prior to behavioral training. Additional studies with the A_2A_R antagonist administration 30 min prior to or post training might better dissociate the learning from motor effect of A_2A_R antagonist.

### Pharmacological Blockade of A_2A_R at the Coding, Consolidation and Expression Phases of Instrumental Behavior Exerted Its Enhanced Effect on Goal-Directed Valuation but Not on Action-Outcome Contingency

To further determine the modulatory effect of A_2A_R on the distinct processes of instrumental behavior (i.e., learning/coding, consolidation and expression phases), we administered KW6002 at specific time course of instrumental learning processes. Based on our previous study showing the effective biological (i.e., motor) effect of KW6002 5 mg/kg maintained for 150–170 min (Shen H.Y. et al., 2008; Yu et al., 2008), we selected the specific three time points for KW6002/vehicle administration (**Figures 2A**, **3A**): (a) prior to training (30 min before RI training) or (b) post training (10 min after RI training) or (c) prior to behavioral testing (30 min before devaluation/omission test but not available in the RI training sessions) to determine the modulatory effects of KW6002 on coding and consolidation phases as well as the expression of instrumental behavior, respectively.

**Figure 2B** shows that KW6002 treatment either at the prior to-training phase or post-training phase did not affect the performance of mice during the RI sessions (main effect between drug administration groups, *F*_2,21_ = 0.536, *p* = 0.593 and training sessions X drug administration groups interaction effect, *F*_14,147_ = 2.480, *p* = 0.108). In the first devaluation test (**Figure 2C**), mice with vehicle injection formed a stable habitual behavior (*F*_1,7_ = 1.787, *p* = 0.223) as expected. Importantly, mice injected with KW6002 prior to everyday RI training session, which is the coding period, decreased their lever presses rate remarkably in the devalued condition (*F*_1,7_ = 8.779, *p* = 0.021), indicating blockade of A_2A_R enhanced goal-directed coding. However, since KW6002 post-training group did show some trend in decreasing lever pressing rate in the devaluation test, albeit not reaching statistical significance (*F*_1,7_ = 0.417, *p* = 0.539), we further explore the goal-directness promoting effect by KW6002 in the consolidation phase, by proceeding a 2-day complementary RI60 training sessions after the first devaluation test. Then we performed second devaluation test as illustrated in **Figure 2A**. After 2 additional days of RI training, both prior to-training and post-training groups significantly reduced lever presses in the devalued condition (prior to-training group, *F*_1,7_ = 6.931, *p* = 0.034; post-training group, *F*_1,7_ = 13.413, *p* = 0.008), i.e., goal-directed behavior, while control group (i.e., injected with vehicle) showed the characteristics of habitual behavior (*F*_1,7_ = 0.552, *p* = 0.482) (**Figure 2D**). Thus, KW6002 treatment in the consolidation phase of instrumental behavior promoted goal-directed behavior as well. Lastly, we performed the omission test during which the established lever press-reward association was reversed, so reward delivery depended on withholding the lever press action. As illustrated in **Figure 2E**, all mice decreased lever presses rate indistinctively in the omission test. Neither interaction effect of testing time X drug administration groups (*F*_10,105_ = 1.124, *p* = 0.359) nor main effect between drug administration groups (*F*_2,21_ = 0.997, *p* = 0.836) were detected. Thus, blockade of A_2A_Rs at the coding or consolidation phases of instrumental behavior enhanced goal-directed valuation but did not affect action-outcome association.

We then sought to investigate whether A_2A_R exerted its effect by acting on expression phase of instrumental behavior. In this experiment, KW6002 was administered 30 min before behavioral tests (devaluation and omission tests), but unavailable in all of the RI training sessions (**Figure 3A**). As expected, both pre-manipulation groups gradually increased lever presses rate and reached the platform and didn’t show any difference between each other (between groups effect, *F*_1,13_ = 0.395, *p* = 0.541; interaction effect of training sessions X pre-manipulation groups, *F*_5,65_ = 0.554, *p* = 0.608) (**Figure 3B**). As **Figure 3C** shows, mice with KW6002 treatment at the expression phase displayed markedly sensitivity to outcome devaluation (*F*_1,6_ = 10.857, *p* = 0.017) compared with the controls (*F*_1,7_ = 0.150, *p* = 0.710) in the devaluation test. Thus, blockade of A_2A_R facilitated expression of goal-directed behavior. In the omission test (**Figure 3D**), both groups decreased their lever presses gradually over testing time (testing time main effect: *F*_5,65_ = 4.226, *p* = 0.020), indicating the timing effectiveness of the omission test. But the tendencies of lever-press decrease rate for the two groups were parallel as indicated by the absent of the drug treatments X testing time interaction effect (*F*_5,65_ = 0.365, *p* = 0.728), though mice injected with KW6002 apparently pressed more than that of the vehicle-treated mice (between-subject effect of drug treatments, *F*_1,13_ = 3.369, *p* = 0.089). The increased lever presses rate by KW6002 in the omission test might attribute to general motor but not learning effect of A_2A_R antagonist, for drug administration was 30 min before the test. Therefore, the action-outcome contingency may not be affected by A_2A_R antagonist.

### Pharmacological Blockade and Genetic Knockout of A_1_Rs Did Not Affect Acquisition or Goal-Evaluation of Instrumental Behavior

**FIGURE 4:**
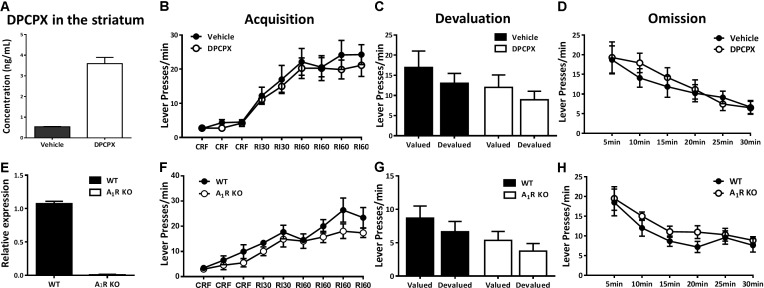
Pharmacological blockade and genetic knockout of A_1_Rs did not affect action-outcome association or goal-evaluation of instrumental behavior. **(A)** The concentration of DPCPX was detected in the striatum of mice 30 min after drug administration (*n* = 3/group), demonstrating the effectiveness of drug level we used. **(B)** Mice with and without DPCPX manipulation performed analogical learning curves in the acquisition of instrumental conditioning (between-subject effect, *p* = 0.597; drug administration X training interaction effect, *p* = 0.867). **(C)** Both DPCPX (*p* = 0.131) and vehicle (*p* = 0.370) groups displayed insensitive to outcome devaluation. **(D)** There didn’t show any difference between DPCPX and vehicle groups in the omission test (between-subject effect, *p* = 0.724; drug administration X testing time interaction effect, *p* = 0.580). **(E)** The knockout efficiency of A_1_R KO mice was confirmed by qPCR. **(F)** A_1_R knockout did not affect acquisition of instrumental behavior since there lack of main effect of genotypes (*p* = 0.219) and training sessions X genotypes interaction effect (*p* = 0.355). **(G)** A_1_R knockout mice and their littermates did not significantly decrease lever presses rate in the devalued condition (A_1_R KO group, *p* = 0.228; WT group, *p* = 0.263). **(H)** Both groups decreased their lever presses indistinctively in the omission test (genotypes main effect, *p* = 0.239; genotypes X testing time interaction effect, *p* = 0.817). All data was analyzed by two-way ANOVA for repeated measurement.

Adenosine acts on facilitating A_2A_R and inhibitory A_1_R to integrate dopamine, glutamate, and BNDF signaling to modulate synaptic plasticity. We next investigated the possible involvement of A_1_Rs in the modulation of instrumental behavior. To ensure the effective DPCPX drug concentration in the striatum after our A_1_R pharmacological treatment paradigm, we determined the pharmacokinetic characteristic of DPCPX (**Figure 4A**) and showed the effective concentration of DPCPX in accordance with its biological effect as described previously (Baumgold et al., 1992). The A_1_R antagonist DPCPX (6 mg/kg) did not affect lever pressing performance during instrumental training sessions (**Figure 4B**, main effect between drug administration groups, *F*_1,14_ = 0.293, *p* = 0.597; interaction effect of drug administration groups X training sessions, *F*_5,70_ = 0.371, *p* = 0.867). The devaluation test proceeded in drug-free condition (**Figure 4C**) revealed that mice with or without DPCPX treatment responded insensitively to satiety devaluation (DPCPX group, *F*_1,7_ = 2.922, *p* = 0.131; vehicle group, *F*_1,7_ = 0.916, *p* = 0.370). In addition, both groups of mice reduced lever presses indistinguishably in the omission test (**Figure 4D**, main effect between drug administration groups, *F*_1,14_ = 0.129, *p* = 0.724; interaction effect of drug administration groups X testing time, *F*_5,70_ = 0.610, *p* = 0.580).

To further confirm this finding by pharmacological blockade of A_1_Rs, we determined the effect of genetic knockout of the A_1_R on acquisition and goal-evaluation using A_1_R knockout mice and their wild-type littermates. The nearly complete deletion of A_1_Rs was verified by qPCR (**Figure 4E**). All mice, regardless of genotypes, increased their rate of lever pressing during the training sessions (**Figure 4F**) with no significant difference between genotypes (*F*_1,13_ = 1.669, *p* = 0.219) or interaction between training sessions and genotypes (*F*_5,65_ = 1.105, *p* = 0.355). During the devaluation test (**Figure 4G**), both A_1_R KO and WT mice similarly showed insensitive to outcome devaluation (A_1_R KO group, *F*_1,6_ = 1.802, *p* = 0.228; WT group, *F*_1,7_ = 1.483, *p* = 0.263), indicating that their responding was habitual. The omission test (**Figure 4H**) further confirmed the results of pharmacological blockade of A_1_R by genetic knockout approach: there was neither main (genotypes) effect (*F*_1,13_ = 1.521, *p* = 0.239) nor the interaction of genotypes X testing time (*F*_5,65_ = 0.260, *p* = 0.817). This finding suggested that A_1_R exerted limited effect on the control of instrumental behavior.

## Discussion

### A_2A_R Antagonist Modulate Animals’ Sensitivity to Goal-Directed Valuation Without Modifying Action-Outcome Contingency

Action-outcome contingency and goal-directed valuation are two cognitive components involved in instrumental conditioning (Balleine and Dickinson, 1998). Action-outcome contingency is determined by the causal relationship between the particular actions and outcomes, while goal-directed valuation depends on the anticipation or desire for the outcome (Yin and Knowlton, 2006). Both components were acquired in the training sessions of instrumental behavior. Thus, outcome devaluation procedure was specialized to probe the importance of the evaluative component of goal-directed actions. We found that pharmacological blockade of A_2A_Rs critically promoted animals’ sensitivity to outcome value (by the devaluation test) but did not affect action-outcome relationship (as manifested by similar performance in the training sessions and in the omission test). When administering 5 min prior to the training, KW6002 at 5 mg/kg apparently elevated the acquisition of learning curve. This enhancement is, however, potentially confounded by the enhanced general motor activity effect of the A_2A_R antagonist. Additional studies with the A_2A_R antagonist administering 30 min prior to or post-training can better dissociate the learning process from motor effect and clarify this issue. The selective modulation of animals’ sensitivity to outcome devaluation by A_2A_R antagonist is in agreement with our recent finding that optogenetic activation of striatopallidal A_2A_R signaling in DMS alters goal-valuation as evident by the devaluation test (Li et al., 2016). On the other hand, the lack of the effect of A_2A_R antagonist on the acquisition of instrumental behaviors collaborates with similar findings by genetic inactivation of striatal A_2A_Rs (Yu et al., 2009) and optogenetic activation of striatopallidal A_2A_R signaling (Li et al., 2016).

The mechanism underlying the selective modulation of goal-valuation by the A_2A_R is not clear. The previous study that overexpression of the D_2_R in the striatopallidal pathway is associated with a shift in behavioral control from habitual action to goal-directed responding but did not affect acquisition phase of instrumental learning (Kwak et al., 2014). Also, loss of striatal endocannabinoid-mediated long-term depression selectively in DLS striatopallidal neurons prevent the transition from goal-directed seeking to habitual responding behavior but did not interfere lever-press performance in the acquisition phase (Gremel et al., 2016). Given the documented antagonistic interaction of the A_2A_R-D_2_R and the A_2A_R-CB_1_R in the striatum by possibly the A_2A_R-D_2_R heterodimers (He et al., 2016) and A_2A_R-CB_1_R heterodimers (Moreno et al., 2017), these findings suggest that A_2A_R may selectively influence coding of the current value of the outcome (but not the contingency association) by the A_2A_R interaction with the D_2_R and CB_1_R functions in the striatum.

Moreover, this selective control of animals’ sensitivity to reward valuation by A_2A_Rs might be related to a motivation factor, as A_2A_R (Mingote et al., 2008; Nam et al., 2013a) and D_2_R (Trifilieff et al., 2013) activities in the striatum contribute to motivational control of behaviors. Lastly, since the A_2A_R are predominantly expressed in the striatopallidal neurons, the A_2A_R control of goal-directed valuation is further supported by the finding from the striatal circuit studies showing that as pharmacogenetic inactivation of the striatopallidal pathway enhanced motivation by energizing the initiation of goal-directed behavior (Carvalho Poyraz et al., 2016), while optogenetic stimulation of the striatopallidal pathway suppressed motivational behavior (O’Hare et al., 2016; Vicente et al., 2016).

### A_2A_R Antagonist Acts at the Coding, Consolidation and Expression Phases of Instrumental Learning to Promote Goal-Directed Behavior

Defining the specific information processing phases (i.e., learning/coding, consolidation and expression of instrumental behaviors) for A_2A_R antagonist control of goal-directed versus habitual behaviors is critical for our understanding of the neurotransmitter modulatory mechanisms and for the development of effective pharmacological strategy to control aberrant habit formation and drug addiction. Our demonstration of the enhanced goal-directed behavior by administration of KW6002 at the pre-training or post-training or expression phases suggests that A_2A_R acts at the coding, consolidation and expression phases of instrumental learning to promote animals’ sensitivity to goal-directed valuation. It should be noted that the influence of the pre-training treatment paradigm on the goal-directed behavior might be partly attributed to its effect on the consolidation phase due to the relatively long-lasting effect (>2 h) of the A_2A_R antagonist KW6002. The similar control of instrumental behaviors by multiple treatment paradigms of KW6002 indicate that A_2A_R control of instrumental behaviors is largely independent of the confounding motor activity.

Various neurotransmitter systems have been implicated in control of the distinct phases of instrumental conditioning. For example, NMDA receptor signaling preferentially affected the coding (by administering NMDA antagonist at the pre-training phase) but not the expression (by administering NMDA antagonist at the post-training phase) of the instrumental conditioning (Yin et al., 2005). Furthermore, virus-induced overexpression of D_2_R (Trifilieff et al., 2013) and 5-HT_6_ receptor (Eskenazi and Neumaier, 2011; Eskenazi et al., 2015) preferentially affect the coding course of operant conditioning. Additionally, optogenetic activation of endocannabinoid signaling in the training session and pharmacogenetic suppression of endocannabinoid signaling in the devaluation test gated habit formation (Gremel et al., 2016), indicating that endocannabinoid modulated instrumental learning in both coding and expression sessions, consistent with the CB_1_R knockout study (Hilario et al., 2007). Thus, the A_2A_R may interact with multiple neurotransmitter systems in the cortico-striatal projection pathways to integrate/modulate glutamate, dopamine and endocannabinoid signaling for instrumental behavioral control at multiple phases of information processing. Furthermore, cognitive control and working memory processes are important for the efficient control of goal-directed behavior (Buschman and Miller, 2014). We and others have documented that the A_2A_R antagonists or focal A_2A_R knockdown in the DMS significantly enhance working memory (Wei et al., 2014; Kaster et al., 2015; Li et al., 2018). Thus, it is possible that when KW6002 is administered prior to the training phase, the A_2A_R antagonist may enhance goal-directed behavior by improving working memory. On the other hand, other mechanisms (such as “off-line” processing during sleep) may contribute to the A_2A_R antagonist-mediated enhancement of goal-directed behavior when A_2A_R antagonists are administered after the training or during the expression/retrieval phase.

### Pharmacological Blockade and Genetic Knockout of A_1_Rs Did Not Affect Acquisition or Goal-Evaluation of Instrumental Behavior

Adenosine signaling acts at the facilitating A_2A_R and inhibitory A_1_R to exert its homeostatic control of brain function. However, very limited information is available regarding the A_1_R control of cognition, particularly instrumental behaviors. With its relatively high expression in the cerebral cortex, hippocampus and striatum (Reppert et al., 1991; Dixon et al., 1996), A_1_R activation has a profound inhibitory control of excitatory transmission by presynaptic and post-synaptic mechanisms (Dunwiddie and Masino, 2001; Ribeiro et al., 2002). Striatal A_1_Rs can preferentially interact with the striatal D_1_Rs via possible A_1_R-D_1_R heterodimers in the striatonigral neurons to control striatal signaling and behavior (Gines et al., 2000). Accordingly, A_1_Rs modulate striatal synaptic plasticity, and prevent scopolamine- and morphine-induced impairment in working memory (Hooper et al., 1996; Lu et al., 2010). However, in the fix-interval and fix-ratio operant training paradigms, A_1_R antagonist failed to increase lever pressing rate, but decreased fix ratio 20 (FR20, every 20 lever presses resulted in one reward) responding at higher doses (Randall et al., 2011). Operant performance alone was insufficient to define instrumental learning modes as goal-directed or habitual actions without devaluation and omission test (Yin and Knowlton, 2006). Thus, the role of the A_1_R in goal-directed versus habitual behaviors is still unknown. Our study demonstrated that pharmacological blockade or global knockout of A_1_R did not affect the acquisition of instrumental learning or sensitivity to reward value or reversal of action-outcome relationship. This finding is in agreement with a recent study that DPCPX failed to reverse the effect of D_2_R antagonist on effort-relevant tasks but KW6002 and caffeine (a non-selective adenosine antagonist) can (Salamone et al., 2009). These findings suggest that A_1_R plays limited modulatory role in control of instrumental behavior and adenosine predominantly acts on A_2A_Rs but not A_1_Rs to modulate instrumental learning.

In summary, our study demonstrated that pharmacological blockade of A_2A_R but not A_1_R promote goal-directed behaviors by enhancing goal-directed valuation without affecting the action-outcome contingency and by acting at the coding, consolidation, and expression phases of goal-directed learning processes. These findings collaborates with our previous genetic and optogenetic studies, and with recent pharmacological studies of A_2A_R antagonists to control abnormal instrumental behavior in drug addiction paradigms (Nam et al., 2013a; Pintsuk et al., 2016), providing pharmacological evidence for a therapeutic strategy to enhance goal-directed behaviors in neuropsychiatric disorders. The translational potential of A_2A_R antagonists is further enhanced by the recent demonstration of the safety profiles of the A_2A_R antagonist KW6002 in clinical phase III trials for motor benefit in >3500 Parkinson’s disease patients (Chen et al., 2013) and by regular consumption of caffeine (a non-specific adenosine A_2A_R and A_1_R antagonist) by 50% world population.

## Author Contributions

YL, YH, XZ, and J-FC designed the experiments. YL, XP, YH, YR, LH, ZW, and CH collected the data. YL, XP, YH, YZ, and ZH analyzed the data. YL, XZ, and J-FC wrote the manuscript.

## Conflict of Interest Statement

The authors declare that the research was conducted in the absence of any commercial or financial relationships that could be construed as a potential conflict of interest.
